# NOTCH and EZH2 collaborate to repress *PTEN* expression in breast cancer

**DOI:** 10.1038/s42003-021-01825-8

**Published:** 2021-03-09

**Authors:** Kyrie Pappas, Tiphaine C. Martin, Andrew L. Wolfe, Christie B. Nguyen, Tao Su, Jian Jin, Hanina Hibshoosh, Ramon Parsons

**Affiliations:** 1 Department of Oncological Sciences, Icahn School of Medicine at Mount Sinai, New York, NY USA; 2 The Tisch Cancer Institute, Icahn School of Medicine at Mount Sinai, New York, NY USA; 3 Department of Pharmacology, Columbia University Medical Center, New York, NY USA; 4 Department of Pathology and Cell Biology, Columbia University Medical Center, New York, NY USA; 5 Mount Sinai Center for Therapeutics Discovery, Department of Pharmacological Sciences, Icahn School of Medicine at Mount Sinai, New York, NY USA; 6 Present Address: Human Oncology and Pathogenesis Program, Memorial Sloan Kettering Cancer Center, New York, NY USA; 7 Present Address: Helen Diller Family Comprehensive Cancer Center, University of California San Francisco, San Francisco, CA USA

**Keywords:** Breast cancer, Breast cancer, Tumour-suppressor proteins

## Abstract

Downregulation of the PTEN tumor suppressor transcript is frequent in breast cancer and associates with poor prognosis and triple-negative breast cancer (TNBC) when comparing breast cancers to one another. Here we show that in almost all cases, when comparing breast tumors to adjacent normal ducts, PTEN expression is decreased and the PRC2-associated methyltransferase EZH2 is increased. We further find that when comparing breast cancer cases in large cohorts, EZH2 inversely correlates with PTEN expression. Within the highest EZH2 expressing group, *NOTCH* alterations are frequent, and also associate with decreased PTEN expression. We show that repression of *PTEN* occurs through the combined action of NOTCH (*NOTCH1* or *NOTCH2*) and *EZH2* alterations in a subset of breast cancers. In fact, in cases harboring *NOTCH1* mutation or a *NOTCH2* fusion gene, NOTCH drives EZH2, HES-1, and HEY-1 expression to repress *PTEN* transcription at the promoter, which may contribute to poor prognosis in this subgroup. Restoration of PTEN expression can be achieved with an EZH2 inhibitor (UNC1999), a γ-secretase inhibitor (Compound E), or knockdown of *EZH2* or *NOTCH*. These findings elucidate a mechanism of transcriptional repression of *PTEN* induced by *NOTCH1* or *NOTCH2* alterations, and identifies actionable signaling pathways responsible for driving a large subset of poor-prognosis breast cancers.

## Abstract (web summary)

Pappas et al. show that the combination of NOTCH and EZH2 alterations drive transcriptional repression of *PTEN* through reversible epigenetic modification of the *PTEN* promoter. These results suggest an actionable target for treating poor-prognosis breast cancer.

## Introduction

Phosphatase and tensin homolog deleted on chromosome ten (PTEN) is a haploinsufficient, dosage-sensitive tumor suppressor that is commonly inactivated or downregulated in cancer. Although genetic mutation of *PTEN* is frequent across many cancer types^1,2^, the loss of PTEN activity in cancer more often occurs in the absence of mutation through complex mechanisms including epigenetic transcriptional repression, microRNAs, noncoding RNAs, and posttranslational modifications, among others^3–7^. In fact, just a 20% decrease in PTEN levels is sufficient to develop breast tumors, and the progressive reduction of PTEN levels is associated with increasingly aggressive tumor phenotypes^8–10^.

Transcription of *PTEN* can be both positively and negatively regulated by a wide variety of transcription factors and chromatin modifying complexes. *PTEN* has been documented to be transcriptionally activated by peroxisome proliferation-activated receptor γ (PPARγ), early growth-regulated transcription factor-1 (EGR1), p53, and activating transcription factor 2 (ATF2)^11–15^. Conversely, *PTEN* has been shown to be transcriptionally repressed by c-Jun and nuclear factor kappa-light-chain-enhancer of activated B cells (NF-κB)^16,17^. Two transcription factors, Snail and inhibitor of DNA binding (ID1), can compete for binding with p53 on the *PTEN* promoter to repress *PTEN* transcription^18,19^. Interestingly, NOTCH can regulate the expression of PTEN through opposing mechanisms depending on the context. Constitutively active NOTCH1 can induce expression of *PTEN* through the MYC and CBF-1 transcription factors in embryonic kidney cells^20,21^ and through direct binding of the Notch intracellular domain to the *PTEN* promoter in endothelial cells^22^, and can repress *PTEN* through binding of the HES-1 transcription factor to the *PTEN* promoter in T cells^23^. The polycomb repressive complex 2 (PRC2) binds chromatin and represses *PTEN* transcription in nasopharyngeal epithelial cells and leukemia through enhancer of zeste homolog 2 (EZH2)-mediated trimethylation of histone 3 lysine 27 (H3K27Me3) at the *PTEN* promoter^24,25^, and is reported to be guided to the site of action at the *PTEN* locus by long noncoding RNA (lncRNA) originating from the *PTENP1* pseudogene locus in certain contexts^26^. Furthermore, histone deacetylases (HDACs) can also restrain *PTEN* expression^27^.

In breast cancer, downregulation of PTEN occurs frequently, especially in poor-prognosis triple-negative breast cancer (TNBC) without any evidence of genetic alteration of the *PTEN* locus in most cases^28,29^. In fact, *PTEN* expression is diminished in 19% of all breast cancers, and in over 50% of TNBCs (RNA-seq z-score cutoff for downregulation < −1)^30,31^, where PTEN transcript level rather than mutation or posttranslational modification is the primary determinant of PTEN protein expression^28^. Baseline expression of p53 also controls PTEN expression in breast cancer, where p53 mutation is associated with decreased expression^15^. *NOTCH1* and *NOTCH2* mutations occurring in breast cancer are required for tumor viability but the signaling pathways through which they maintain tumor growth are similarly unclear^32^.

To clarify the mechanisms responsible for the silencing of PTEN expression, we carried out an analysis of primary breast cancer samples, adjacent normal epithelial tissue, and existing breast cancer datasets for genes that could be responsible for PTEN loss of expression and determined that *NOTCH1* or *NOTCH2* alterations (mutation, fusions, or overexpression) occur in a large proportion of TNBC cases exhibiting PTEN downregulation. Using breast cancer cell lines, we found that mutant forms of NOTCH1 or NOTCH2 collaborate with EZH2 to mediate the transcriptional repression of *PTEN* in these poor-prognosis breast cancers. PTEN expression could be restored by interfering with NOTCH or EZH2 function, thus highlighting a therapeutic strategy for these patients.

## Results

### The PTEN locus is transcriptionally repressed in breast tumors relative to normal breast tissue

**Fig. 1:**
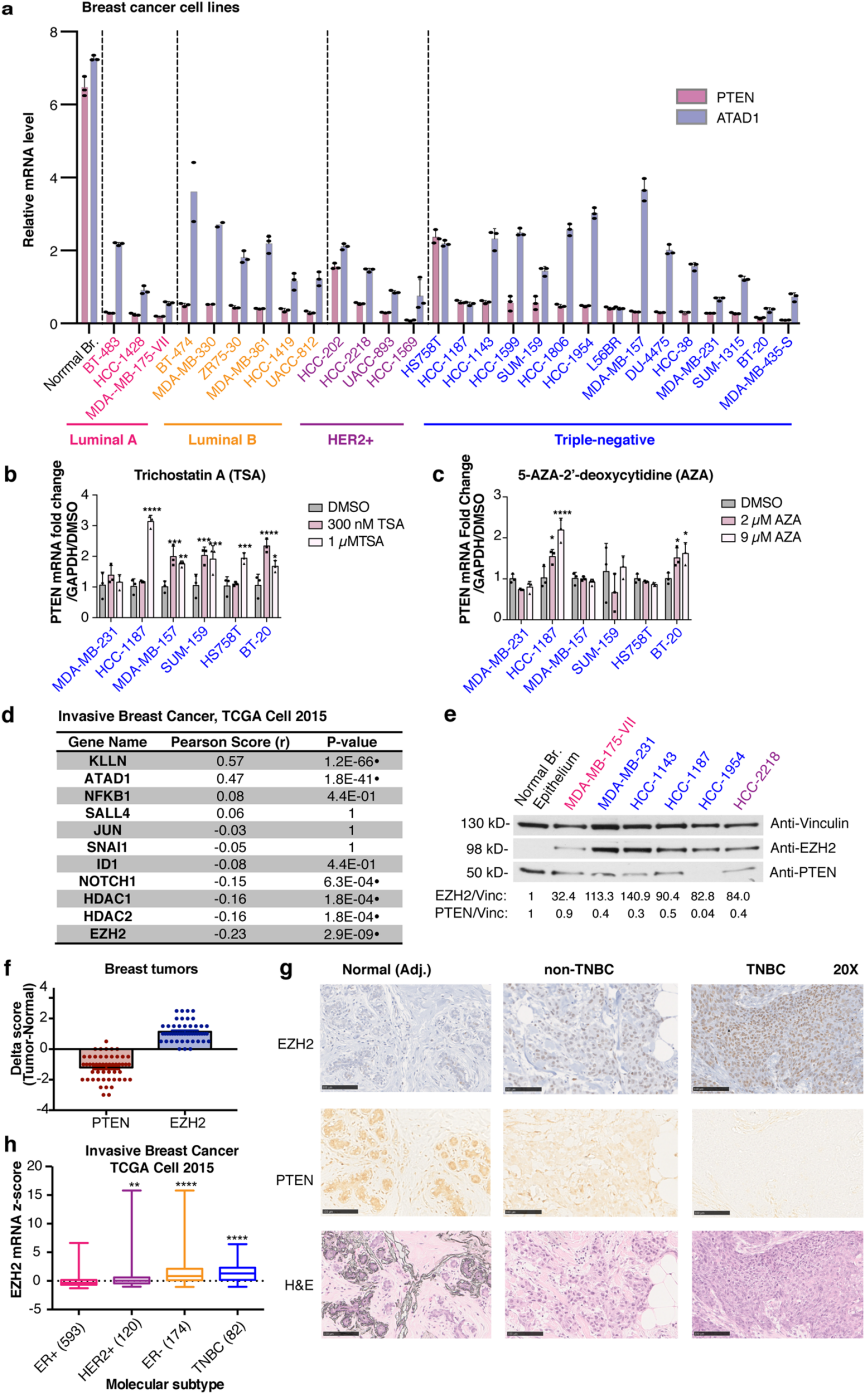
Repression of the *PTEN* locus occurs in breast cancer, and associates with increased expression of *EZH2* and other known transcriptional regulators. **a** *PTEN* (red) and *ATAD1* (blue) transcript levels were measured using qRT-PCR in breast cancer cell lines compared to normal mammary epithelial cells. Error bars are mean ± s.d., triplicate measurements. Significance from normal mammary epithelial cells derived from normal mammoplasty specimen: two-way ANOVA, Dunnett’s correction (*P* < 0.0001 for all). **b** Trichostatin A (TSA) and (**c**) 5-aza-2′-deoxycytidine (AZA) treatment was performed at the indicated doses and transcript levels of PTEN were measured by qRT-PCR in breast cancer cell lines. Error bars: mean ± s.d., triplicate measurements. Significance from Ctrl: one-way ANOVA, Sidak’s correction. **d** Analysis of co-expression with *PTEN* including Pearson correlation coefficients and *P* values in normalized RNA-seq data for previously reported *PTEN*-repressing genes and genes in the *PTEN* genomic locus (ATAD1 and KLLN). Cohort includes 818 breast cancer cases^34^. Significance: two-tailed *t* test, Pearson correlation (*n* = 818), black dot indicates a statistically significant result. *P* values adjusted for multiple comparisons by the Benjamini and Yekutieli method, *α* = 0.001^62^. **e** Protein levels of EZH2 and PTEN were measured by immunoblotting in the panel of indicated cell lines. Vinculin was loading control. Quantification is shown below each blot (signal normalized to Vinculin, calculated as a fold change compared to normal breast epithelium isolated from normal mammoplasty specimen). **f** Immunohistochemical analysis of breast tumors showing PTEN and EZH2 score. Score is expressed as a delta between tumor and normal ducts on the same slide (*n* = 51 cases). Clinical scoring is used (0–3+ range based on intensity). Error bars: mean ± s.e.m. **g** Reperentative photos of IHC staining in normal ducts (BRP ID# 6621), non-TNBC (BRP ID# 6617), and TNBC (BRP ID# 6706), showing PTEN, EZH2 and H&E. ×5 magnification, Scale bar: 500 µm. **h** Boxplots of *EZH2* RNA-seq *z*-scores in the indicated subtypes of breast cancer^34^, number of cases indicated for each subtype (total *n* = 969). Significance from ER+: one-way ANOVA, Dunnett’s correction. (****P < 0.0001; ***P < 0.001; **P < 0.01; *P < 0.05).

Many previous measurements of PTEN mRNA have been based on a comparison among tumors without regard to the normal level of expression. However, PTEN mRNA measurements correlate well with protein levels measured by immunohistochemistry (IHC)^28^, which are scored relative to normal epithelium in the same section. To better understand the relationship between normal mammary epithelial tissue and breast tumor tissue expression, and to potentially identify cell culture models for studying PTEN downregulation, we measured expression of PTEN in epithelial cells isolated from normal breast and a large series of breast cancer cell lines that were genetically wild-type for *PTEN*^33^. PTEN transcript was downregulated in breast cancer cell lines compared to normal mammary epithelial cells in breast cancers of all subtypes, and the same was true regarding the neighboring gene *ATAD1*, that shares an enhancer with *PTEN*, and is part of the PTEN-loss signature (Fig. 1a, Supplementary Table 1)^28^. mRNA levels of ATAD1 are highly correlated with PTEN mRNA levels in a large cohort of breast cancers of all subtypes (*P* < 0.0001, Supplementary Fig. 1a)^30,31^. These data suggest that breast tumor cell lines could be potential models for studying the downregulation of PTEN by epigenetic regulation of the neighborhood of chromatin including *PTEN*, and also suggest that PTEN is more frequently downregulated than previously realized.

To determine if the PTEN mRNA downregulation in tumors relative to normal was also present in patient samples, we used Nanostring digital barcoding technology to measure PTEN expression in normal and tumor breast biopsy samples (four normal samples, four normal–tumor pairs) using multiple different probes, and determined that PTEN levels are decreased in tumor compared to normal samples (Supplementary Fig. 1b, Supplementary Data 1, Supplementary Table 2). Furthermore, PTEN-downregulated samples were associated with changes in the expression of selected previously published PTEN-loss signature genes including *ATAD1* (Supplementary Fig. 1c and Supplementary Data 1), indicating agreement between the breast cell lines and patient samples.

### PTEN expression inversely correlates with EZH2 and NOTCH1

To explore the cause of loss of PTEN expression in breast cancer, we treated breast cancer cell lines with the epigenetic inhibitors Trichostatin A (TSA), which inhibits Class I and II histone deacetylases (HDACs), and 5-aza-2′-deoxycitidine (AZA) which is a cytidine analog that blocks DNA methylation (Fig. 1b, c). We saw substantial restoration of PTEN transcript levels in multiple cell lines following TSA treatment, with the exception of MDA-MB-231 (Fig. 1b). This restoration was not as apparent with AZA treatment, where only HCC-1187 and BT-20 cells showed an increase in PTEN transcript upon treatment (Fig. 1c). We assessed potential regulators of PTEN for their association with PTEN mRNA in a large cohort of breast cancer cases from The Cancer Genome Atlas (TCGA)^30,31,34^. Interestingly, EZH2, the histone lysine methyltransferase component of the PRC2 complex, had the largest inverse correlation with PTEN expression in a large cohort of breast cancer cases, and the transcriptional regulator NOTCH1 was also inversely correlated with *PTEN* expression (Fig. 1d, Supplementary Data 2 for all genes). Furthermore, the expression of HDACs (HDAC1 and HDAC2) that interact with the PRC2 complex was also negatively correlated with PTEN expression (Fig. 1d, Supplementary Data 2), consistent with the result of TSA treatment in breast cancer cell lines. The expression of the *ATAD1* and *KLLN* genes that are in genomic proximity to the *PTEN* locus were strongly positively correlated with PTEN expression, as previously reported^28^, again suggesting a common epigenetic mechanism of regulation controlling the region (Fig. 1d, Supplementary Data 2).

To characterize a subset of seven cell lines further, we performed immunoblot measurements of PTEN from protein lysates and confirmed that PTEN protein levels reflect the change in mRNA expression (Fig. 1e, Supplementary Fig. 1d). In a larger cohort of 841 breast tumors, PTEN transcript and protein levels are highly correlated (*P* < 0.0001, Supplementary Fig. 1e)^30,31^. Furthermore, we show that EZH2 protein levels are increased in breast cancer cell lines compared to normal breast epithelial cells (Fig. 1e, Supplementary Fig. 1d). We measured PTEN and EZH2 protein expression by IHC in breast cancer cases, and we observed the same inverse correlation (Fig. 1f, g, Supplementary Data 3). Generally, PTEN staining decreases in tumor versus adjacent normal, and EZH2 staining increases, though the magnitude of the changes between tumor and normal varies between cases (Fig. 1f). These results strengthen the observation that PTEN downregulation is exceptionally frequent in all subtypes of breast cancer (Supplementary Data 3). Adjacent normal ducts typically have low/no EZH2 staining and robust PTEN protein levels (Fig. 1g). We also observed that EZH2 levels in tumor (compared to normal) tend to be higher in TNBC cases (Supplementary Data 3). In fact, in a larger cohort of breast cancer cases^34^, we found that EZH2 mRNA expression is increased in more aggressive subtypes of breast cancer including TNBC (Fig. 1h)^30,31,34^. Thus, we decided to investigate a putative PRC2-based mechanism by which PTEN may be transcriptionally downregulated in breast cancer cell lines and primary tumors.

### The PTEN promoter contains repressive domains that are prominent in some breast cancer cell lines

**Fig. 2:**
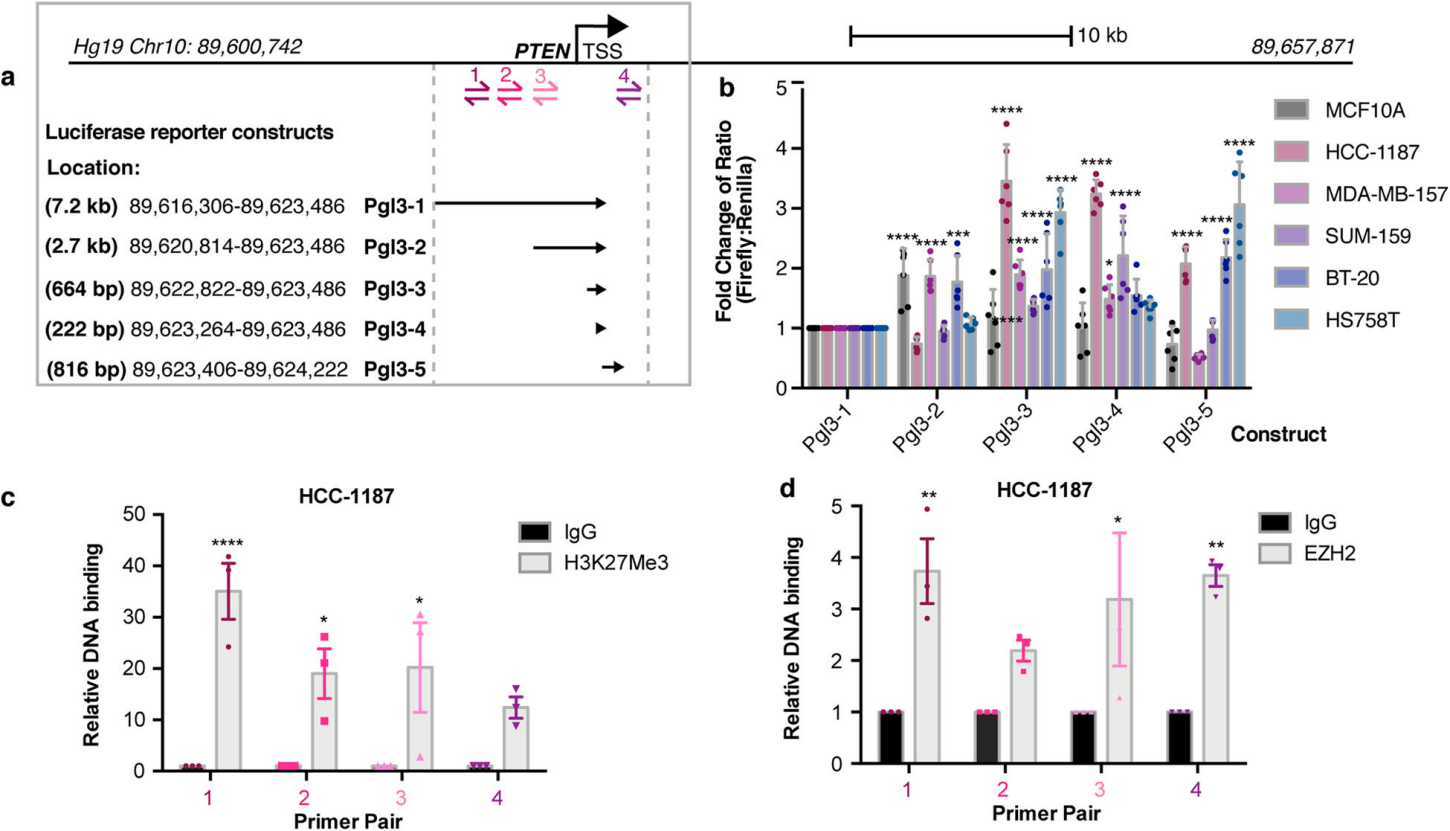
Mapping transcriptional repression of the *PTEN* promoter. **a** Map of the *PTEN* promoter and upstream regulatory region where primer sites for ChIP-qPCR are shown (Sites 1–4). Location of luciferase reporter constructs^23^, Hg19 genomic loci on Chr.10, and length in base pairs listed. **b** Luciferase activity normalized to Renilla for each luciferase construct calculated as a fold change from Pgl3-1 construct. Cell line is indicated. Error bars are mean ± s.d. (mean of six measurements: two biological replicates, triplicate measurements each). Significance from Pgl3-1: two-way ANOVA, Dunnett’s correction. **c**, **d** ChIP-qPCR in HCC-1187 cells for (**c**) H3K27Me3 and (**d**) EZH2 at indicated regions (1, 2, 3, 4) of the *PTEN* promoter. Relative DNA binding is % input normalized to IgG. Error bars: mean ± s.e.m., *n* = 3 experiments. Significance from IgG: two-way ANOVA, Sidak’s correction. (****P < 0.0001; ***P < 0.001; **P < 0.01; *P < 0.05).

We sought to determine which regions of the *PTEN* promoter are the most important for *PTEN* transcriptional repression in breast cancer. We chose a panel of *PTEN* wild-type cell lines comprised of the non-tumorigenic mammary epithelial cell line MCF10A, and breast cancer cell lines that have low *PTEN* transcript levels, including HCC-1187, MDA-MB-157, SUM-159, BT-20, and HS758T. We performed a luciferase reporter assay using previously defined sections of the *PTEN* promoter/regulatory region that contain transcriptionally active chromatin elements in various non-tumorigenic breast cell lines and tissue (separated by compartment) as well as in breast cancer cell lines (Supplementary Fig. 2) fused to a luciferase reporter gene (Fig. 2a)^23^. Furthermore, this region contains hotspot mutations in breast cancer^35^, suggesting its importance in transcriptional regulation of PTEN (Supplementary Fig. 2). The strength of the luciferase signal corresponds to the transcriptional activity of that section of the *PTEN* promoter. We found that compared to the longest *PTEN* promoter/regulatory region reporter, multiple truncated portions caused increased transcriptional reporter activity in all of the cancer cell lines with a particularly notable increase for HCC-1187, consistent with the full-length *PTEN* promoter containing repressive elements (Fig. 2b). On the other hand, for the non-tumorigenic line MCF10A, only one truncated region (Pgl3-2) led to a relatively modest increase in PTEN transcription, indicating that less active repressive mechanisms may also be present in non-tumorigenic cells (Fig. 2b).

### EZH2 binds to the PTEN genomic locus at regions important for transcriptional repression containing H3K27 trimethylation

We then further investigated epigenetic landscape of the *PTEN* promoter/regulatory region that are important for transcriptional repression in cancer. To do this, we performed chromatin immunoprecipitation (ChIP) coupled with qPCR for H3K27Me3, the mark of PRC2-mediated transcriptional repression, in the HCC-1187 TNBC cell line, which we selected because it is *PTEN* wild-type, exhibited marked co-downregulation of PTEN and ATAD1 (Fig. 1a), and showed the greatest amount of reporter activation when portions of the *PTEN* promoter were deleted (Fig. 2b). Examination of HCC-1187 at the *PTEN* promoter/regulatory region revealed extensive H3K27Me3 (Fig. 2c). Notably, the regions containing interaction with H3K27Me3 overlapped with those that were important for transcriptional repression in the luciferase reporter assay (Fig. 2b). To determine if the PRC2 member EZH2 could also be present in this region, we performed ChIP for EZH2 and observed binding of EZH2 to the *PTEN* promoter/regulatory region in the same regions of chromatin (Fig. 2d). This result suggested that the PRC2 complex acts at the *PTEN* locus and may contribute to the transcriptional repression of PTEN observed in breast cancer

### Depletion of EZH2 restores PTEN expression in cases harboring NOTCH1 or NOTCH2 mutations

**Fig. 3:**
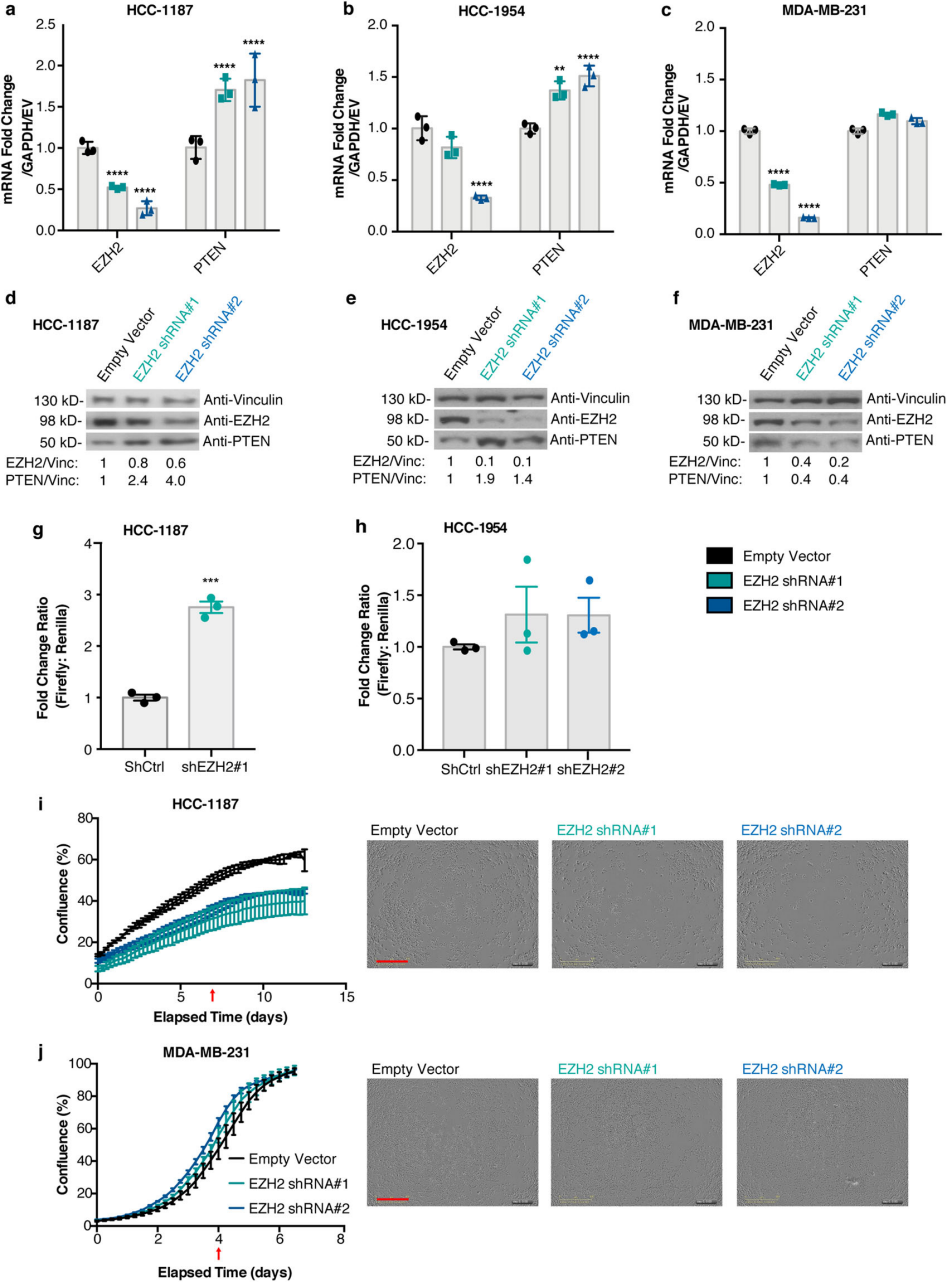
Depletion of EZH2 restores PTEN expression and reduces proliferation in a subset of cell lines. Stable shRNA knockdown of EZH2 was performed. EZH2 and PTEN transcript levels were measured by qRT-PCR (**a**–**c**) and protein levels were measured by immunoblotting in (**d**–**f**) HCC-1187 cells, HCC-1954 cells, and MDA-MB-231 cells, respectively. Vinculin was loading control. Quantification is shown below each blot (signal normalized to Vinculin, calculated as a fold change compared to Empty Vector). Error bars: mean ± s.d. (triplicate measurements). Significance from Ctrl: two-way ANOVA, Tukey’s correction. Luciferase activity at the *PTEN* promoter was measured (Pgl-1 from Fig. 2a) following stable knockdown of *EZH2* in (**g**) HCC-1187 and (**h**) HCC-1954 cells. Error bars: mean ± s.e.m., *n* = 3 experiments. Proliferation was measured following *EZH2* knockdown, the percentage of confluence over time (days) is displayed in (**i**) HCC-1187 cells and (**j**) MDA-MB-231 cells. Readings taken every 6 h. Error bars: mean ± s.d., triplicate measurements. Corresponding representative photos from indicated timepoints (red arrow) shown, Scale bar (red): 800 µm. Significance from Ctrl: two-way ANOVA, Tukey’s correction. (****P < 0.0001; ***P < 0.001; **P < 0.01).

We next wanted to investigate a possible role for EZH2 in the repression of *PTEN*, which we examined in HCC-1187 and two additional cell lines that exhibited low levels of PTEN (in the absence of mutation), HCC-1954, and MDA-MB-231. We performed a stable knockdown of EZH2 in HCC-1187 cells and observed that PTEN transcript and protein levels were restored following knockdown (Fig. 3a, d, respectively, Supplementary Fig. 2b). We saw a similar increase in PTEN transcript and protein levels following EZH2 knockdown in HCC-1954 cells (Fig. 3b, e, respectively) but not in MDA-MB-231 cells (Fig. 3c, f, Supplementary Fig. 2b). Furthermore, transcriptional activity at the *PTEN* promoter was increased upon EZH2 knockdown in both HCC-1187 and HCC-1954 cells (Fig. 3g, h). It has been previously demonstrated that subtle variations in PTEN dose can also influence tumorigenic properties such cell proliferation^9^. The effect of EZH2 knockdown on proliferation was evaluated in HCC-1187 and MDA-MB-231, and EZH2 knockdown decreased proliferation in HCC-1187 cells (Fig. 3i) but did not change proliferation in MDA-MB-231 cells (Fig. 3j), suggesting that the decrease in proliferation in HCC-1187 cells may at least in part be due to the increase in PTEN expression.

**Fig. 4:**
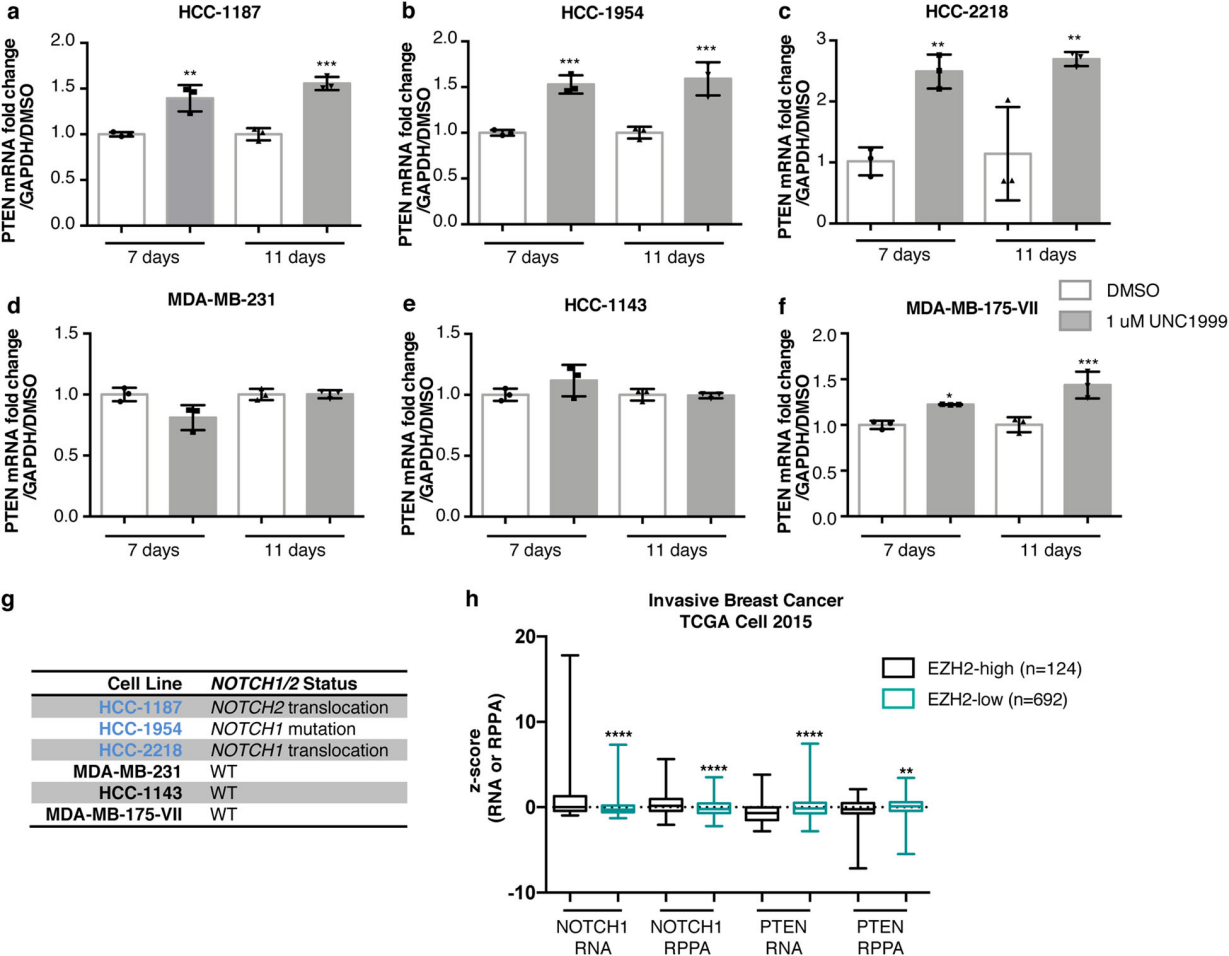
Mutation of *NOTCH1/2* enhances restoration of *PTEN* expression in response to EZH2 inhibition. Inhibition of EZH2 by UNC1999 was performed for 7 days and 11 days and transcript levels of PTEN were measured by qRT-PCR in (**a**) HCC-1187, (**b**) HCC-1954, (**c**) HCC-2218, (**d**) MDA-MB-231, (**e**) HCC-1143, and (**f**) MDA-MB-175-VII. Error bars: mean ± s.d., triplicate measurements. Significance from Ctrl: one-way ANOVA, Sidak’s correction. **g** Table shows the *NOTCH1/NOTCH2* mutation status of breast cancer cell lines^32,40^. **h** Boxplots of NOTCH1 and PTEN RNA and protein levels in EZH2-high (RNA-seq *z*-score > 1) and EZH2-low (RNA-seq *z*-score < 1) breast cancer cases, measured by RNA-seq and RPPA, respectively^34^. Number of cases indicated for each group (total *n* = 816). (****P < 0.0001; ***P < 0.001; **P < 0.01; *P < 0.05).

As S-adenosyl-L-methionine (SAM)-competitive EZH2 inhibitors are highly selective and are currently showing promise in clinical trials for lymphoma and malignant rhabdoid tumors^36^, we decided to test whether the preclinical EZH2 inhibitor UNC1999^37^ could restore expression of PTEN in breast cancer cell lines. UNC1999 restored PTEN expression considerably in the HCC-1187, HCC-1954, and HCC-2218 cell lines, restored it weakly in MDA-MB-175-VII, but did not in the MDA-MB-231 or HCC-1143 cell lines (Fig. 4a–f), and these results were consistent with our EZH2 stable knockdown experiments. Given the reported association between NOTCH and EZH2 in invasive breast cancer^38,39^, we examined the *NOTCH* status of these cell lines and found that the three cell lines that restored PTEN expression considerably in response to EZH2 inhibition or knockdown all harbor mutations or fusions in *NOTCH1* or *NOTCH2*, whereas the other cell lines harbor wild-type *NOTCH1* and *NOTCH2*^32,40^ (Fig. 4g). To determine the relevance of our findings in tissue culture to the downregulation of PTEN that is observed in human breast tumor surgical samples, we examined the large TCGA data cohort containing mRNA and protein expression data and found that cases expressing high levels of EZH2 (RNA-seq *z*-score >1, about 15% of all breast cancers and 57% of TNBCs) tend to have increased expression of NOTCH1 and decreased expression of PTEN at both the transcript and protein levels^30,31,34^ (Fig. 4h). Our previous work identified a p53-dependent enhancer for *PTEN*^15^ (p53 binding site shown in Supplementary Fig. 2), so we hypothesized that a p53 mutation might prime cells for repression mediated by EZH2. Even though we saw a strong association between *TP53* mutation and increased EZH2 transcript levels that has been previously observed^41,42^ (Supplementary Fig. 3a, b), the ability of UNC1999 to restore PTEN levels in cell lines did not correlate with p53 mutation status^30,31,34^ (Supplementary Fig. 3c). Overall, these results show that depletion of EZH2 activity may be effective at restoring repressed *PTEN* in cases harboring *NOTCH* alterations.

### Mutant NOTCH drives both increased expression of EZH2 and transcriptional repression of PTEN in breast cancer

**Fig. 5:**
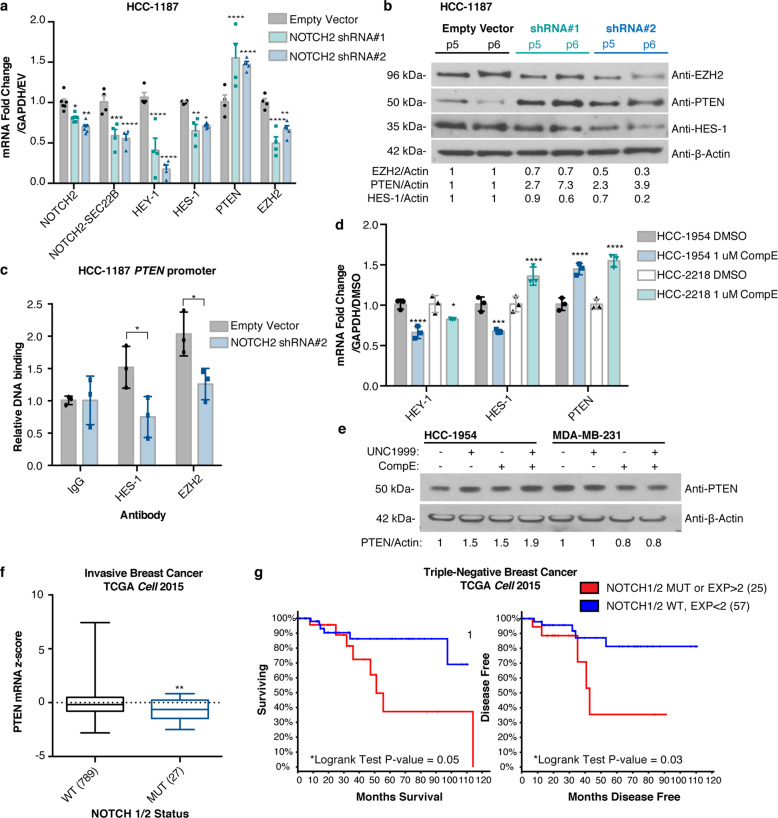
Mutant *NOTCH1/2* collaborates with EZH2 to drive transcriptional repression of PTEN and associates with poor prognosis. **a** Transcript levels measured by qRT-PCR and (**b**) protein levels measured by immunoblotting of indicated genes following stable knockdown of NOTCH2. P5, P6 indicate passage number after infection. β-actin was the loading control and quantification is shown. Error bars: mean ± s.e.m., *n* = 4 experiments. Significance from Ctrl: two-way ANOVA, Dunnett’s correction. **c** ChIP-qPCR binding of indicated proteins to the *PTEN* promoter (Site B^23^, between Sites 3 and 4, Fig. 2a) following stable knockdown of NOTCH2. Relative DNA Binding is % input (normalized to IgG). Significance from Ctrl: two-way ANOVA, Sidak’s correction. Error bars: mean ± s.d., triplicate measurements. **d** HCC-1954 and HCC-2218 cells were treated for 6 days with 1 μM Compound E and expression of indicated genes were measured using qRT-PCR. Error bars: mean ± s.d., triplicate measurements. Significance from Ctrl: 2-way ANOVA, Tukey’s correction. **e** HCC-1954 and MDA-MB-231 cells were treated for 6 days with 1 μM Compound E and/or UNC1999 and expression of indicated genes were measured using immunoblotting. β-actin was the loading control and quantification is shown. **f** Boxplots of PTEN RNA-seq data from invasive breast cancer cases^34^ in *NOTCH1/2* wild-type (WT) or mutant (MUT) groups. Number of cases indicated for each group (total *n* = 816). Significance: Mann–Whitney test. **g** Reduced overall survival and disease-free survival in TNBC cases^34^ harboring *NOTCH1 or NOTCH2* mutations or overexpression (RNA-seq *z*-score > 2). Number of cases indicated for each group (total *n* = 82). Significance: Logrank test. (****P < 0.0001; ***P < 0.001; **P < 0.01; *P < 0.05).

We demonstrated that a subset of breast cancer cell lines exhibit reduced expression of *PTEN* mediated by the PRC2 complex, but we wanted to investigate the possible upstream signaling changes that cause increased PRC2 activity at the *PTEN* promoter. EZH2 can activate NOTCH signaling in breast cancer^38^, mutation of *NOTCH1* can lead to repression of *PTEN* through the HES-1 transcription factor in T cells^23,43^, and *NOTCH1*/2 translocations and mutations in breast cancer can increase activity or create a truncated form of NOTCH resembling cleaved NOTCH that enters the nucleus to regulate transcription of target genes^32,44^. Interestingly, the HCC-1187 cell line, where we detected evidence of EZH2-mediated repression of *PTEN*, harbors a transforming *SEC22-NOTCH2* translocation^32^. To examine the role of NOTCH2 in the repression of *PTEN* in HCC-1187 cells, we performed a stable knockdown of NOTCH2 and observed a restoration of PTEN transcript and protein levels (Fig. 5a, b, Supplementary Fig. 3d). Concomitant with the stable restoration of PTEN expression, we observed a decrease in the NOTCH target genes HEY-1 and HES-1, as well as a decrease in EZH2 transcript and protein levels (Fig. 5a, b, Supplementary Fig. 3d). We performed ChIP-PCR on the *PTEN* locus in HCC-1187 cells and observed that both HES-1 and EZH2 bind directly to the same sites on the *PTEN* promoter (Fig. 5c). Furthermore, HES-1 and EZH2 binding to the *PTEN* promoter was diminished by NOTCH2 knockdown (Fig. 5c).

The HCC-1954 and HCC-2218 cell lines harbor *NOTCH1* mutations (missense and translocation, respectively)^32,40^. To investigate the role of NOTCH1 in the transcriptional repression of *PTEN* in these cell lines, we inhibited downstream NOTCH signaling with the γ-secretase inhibitor Compound E (CompE). In HCC-1954 cells, treatment with CompE caused a decrease in both HES-1 and HEY-1 transcript levels, whereas in HCC-2218 cells, the treatment only inhibited HEY-1 transcript levels (Fig. 5d). This result is consistent with previous research showing that the *NOTCH1* translocation present in HCC-2218 cells signals primarily through HEY-1^32^. Furthermore, treatment with CompE resulted in an increase in PTEN transcript levels in both HCC-1954 and HCC-2218 cell lines (Fig. 5d), and an increase in PTEN protein levels in HCC-1954 cells but not MDA-MB-231 cells (Fig. 5e, Supplementary Fig. 3d), indicating that alterations in *NOTCH1* and *NOTCH2* can contribute to transcriptional repression of PTEN. The combination treatment with CompE and UNC1999 led to an additive restoration of PTEN protein expression compared to either drug alone in HCC-1954 but not MDA-MB-231 cells (Fig. 5e, Supplementary Fig. 3d), further supporting this conclusion.

### NOTCH1 and NOTCH2 alterations are correlated with reduced PTEN expression in breast cancer biopsies and represent a poor-prognosis subset of TNBC

To determine if *NOTCH1* and *NOTCH2* alterations could be regulating PTEN in tumor biopsies, we examined the same TCGA breast cancer cohort that we analyzed for EZH2^30,31,34^, and found that the presence of *NOTCH1* or *NOTCH2* mutations in breast cancer (about 3% of cases) correlated with reduced PTEN expression^30,31^ (Fig. 5f). Furthermore, within TNBC, a subtype of breast cancer harboring high expression of EZH2, combined *NOTCH1* and *NOTCH2* mutation or overexpression (occurring in about 30% of TNBC cases) is associated with decreased overall survival and decreased disease-free survival^30,31,34^ (Fig. 5g), which likely depends on multiple NOTCH outputs including *PTEN*. These results combined with our cell line findings suggest that alteration of *NOTCH1* or *NOTCH2* (mutation or overexpression) could contribute to increased PRC2 complex activity at the *PTEN* promoter in these breast cancers, and may account for a large proportion of the PTEN-downregulated cases in TNBC. The HES-1 and HEY-1 transcription factors could recruit the PRC2 complex to the *PTEN* cis-regulatory elements such as the *PTEN* promoter. Moreover, these results suggest that this signaling activity could be responsible for the poor prognosis for this subset of patients.

## Discussion

**Fig. 6:**
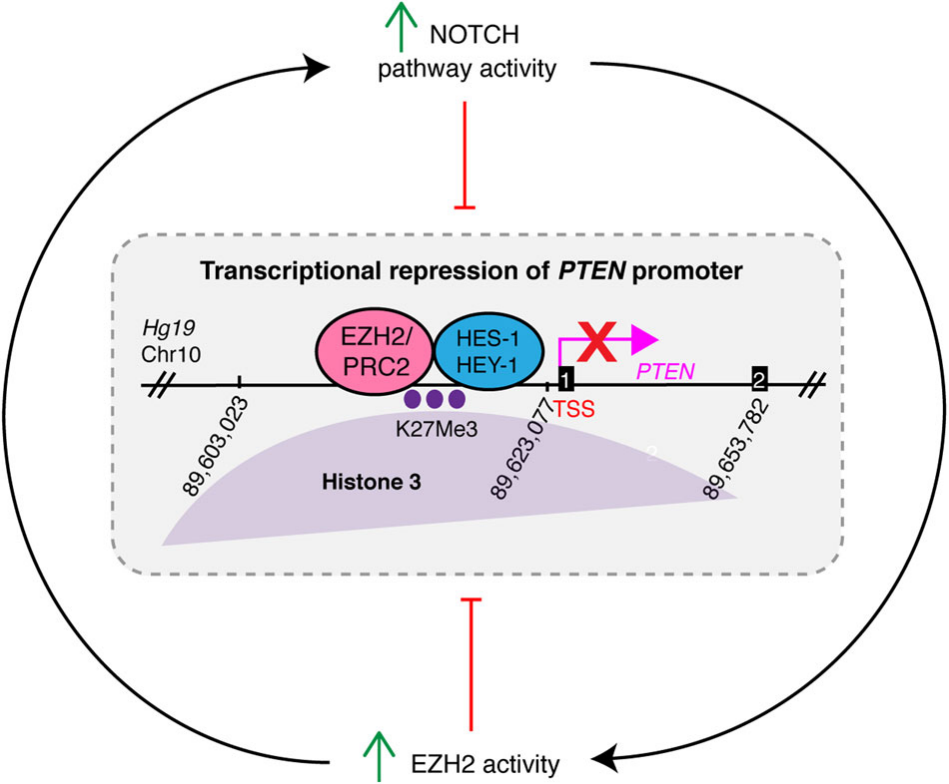
Model of transcriptional repression of *PTEN*. The NOTCH and PRC2/EZH2 pathways form a regulatory loop where both can feed into the transcriptional repression of PTEN. Mutation or translocation of *NOTCH1* or *NOTCH2* leads to upregulation of *HES-1/HEY-1* and PRC2/EZH2; however, *NOTCH* mutation is not required for EZH2 activity or transcriptional repression. EZH2 signaling can also upregulate NOTCH^38^. PRC2/EZH2 adds the H3K27Me3 mark of transcriptional repression (purple dots), and binds with the HES-1 transcription factor on the *PTEN* promoter leading to repression. Numbers 1 and 2 in black boxes represent exons of *PTEN*.

Here, we discover that dysregulation of both PTEN and EZH2 occurs in almost all breast cancers when compared to adjacent normal ducts, regardless of subtype. However, the degree of downregulation of PTEN and upregulation of EZH2 is most severe in TNBC cases compared to other subtypes of breast cancer^30,31,45^. Using genomic profiles of breast tumors and cell lines, we have uncovered a molecular mechanism of transcriptional repression of PTEN in breast cancer. These results suggest that EZH2 and NOTCH1 or NOTCH2 collaborate to mediate the transcriptional repression of PTEN, and that oncogenic alterations in *NOTCH1* and *NOTCH2* may (directly or indirectly) drive the increased expression of *EZH2, HES-1*, and *HEY-1* whose protein products collaborate to repress the *PTEN* promoter (Fig. 6). Previous work has shown that HES-1 inhibits RNA polymerase II-mediated transcription elongation by preventing recruitment of the P-TEFb complex^46^, which could potentially cooperate with the PRC2 complex to facilitate transcriptional repression at the *PTEN* promoter. Based on our observations in Fig. 1, it is also likely that other factors exist that can increase PRC2 activity at the *PTEN* promoter independently of NOTCH1 and NOTCH2 pathway activity, as well as PRC2-independent mechanisms of *PTEN* repression in breast cancer. Importantly, while p53 mutation associates with increased EZH2 z-score, restoration of PTEN transcript in response to EZH2 inhibition does not depend on p53 mutation status.

Increased expression of EZH2 transforms normal breast epithelial cells, is a marker of aggressive breast cancer, and associates with poor prognosis^47,48^ and EZH2 has been shown to expand stem cell populations in breast cancer though activation of the NOTCH1 pathway^38^. It was previously demonstrated that EZH2 promotes metastasis in MDA-MB-231 cells, which can be mitigated by stable knockdown^49^. However, effect of EZH2 knockdown on invasion is more pronounced than its effect on proliferation—indicating that the promotion of metastasis by EZH2 in this particular case is occurring through a different mechanism.

*NOTCH* mutations are oncogenic in mouse models of breast cancer^50,51^, and *NOTCH1* and *NOTCH2* mutations drive tumorigenic cell growth and signaling in human breast cancer^32^. Increased expression of NOTCH1 and EZH2 individually associate with poor prognosis in breast cancer, and their expression levels are positively correlated with one another^39^. Although mutation of *NOTCH1* or *NOTCH2* only occurs in a small proportion of breast cancer cases, inclusion of cases harboring both mutation and overexpression accounts for 30% of poor-prognosis TNBC cases. It has been reported that MDA-MB-231 cells harbor a slight elevation in NOTCH1 protein and thus, are subtly susceptible to a γ-secretase inhibitor (PZ0187), which is cytostatic to subcutaneous MDA-MB-231 xenografts, and has a slight restorative effect on PTEN protein levels^52^. We did not see the same effect on PTEN with a different γ-secretase inhibitor (CompE). These studies combined with our findings support the idea that *NOTCH1* or *NOTCH2* alterations could generate a broadly relevant positively reinforcing feedback loop between the NOTCH and EZH2 pathways that may have important roles in driving a large proportion of the poor-prognosis cancers that display downregulation of PTEN (Fig. 6). Importantly, the action of NOTCH is highly tissue specific; therefore, the insight that NOTCH plays this potentially oncogenic role in the repression of *PTEN* in poor-prognosis breast cancer is of interest to the field. It is possible that cells in the tumor microenvironment can activate NOTCH signaling and downregulate PTEN in a cell non-autonomous manner through juxtacrine signaling to tumor epithelial cells, as has been demonstrated with macrophages and other components of the breast cancer stroma^53,54^. Additionally, ER-negative breast cancer stem cells rely on NOTCH-dependent paracrine signaling from ER-positive cells in the mammary epithelium^55^.

Our findings also suggest that NOTCH1 and NOTCH2-driven breast cancers may represent a distinct biological form of breast cancer that is driven in part through the silencing of the *PTEN* tumor suppressor gene, in addition to the activation of oncogene targets such as MYC^56^. Interestingly, the repression of tumor suppressors by polycomb group (PcG) proteins including PRC2 could be a more broadly relevant mechanism of tumor suppressor repression in cancer. Collaboration between PcG proteins and the NOTCH pathway contributes to malignancy in *Drosophila* through silencing of the retinoblastoma (Rb1) tumor suppressor^57^. In fact, in breast cancer tumor samples, *RB1* expression levels are correlated with PTEN expression, and inversely correlated with EZH2, HDAC1, HDAC2, and NOTCH1 expression (Supplementary Data 4), which suggests that a similar mechanism of repression may be at play for *RB1*. However, correlation does not necessarily imply a mechanistic linkage, and this hypothesis warrants further experimental testing.

A large body of research has demonstrated that PTEN is a haploinsufficient tumor suppressor that is extremely dosage sensitive^8,9^. Thus, the strong transcriptional downregulation of PTEN observed in many types of cancer, including TNBC, could contribute to tumorigenic phenotypes in many cases. Taken together, our results suggest that NOTCH and EZH2, working together in a feed forward loop, could control tumorigenic phenotypes in a subset of breast cancer cases through repression of *PTEN* expression. EZH2 is a promising therapeutic target for many different types of cancer^36^, and our results show that EZH2 inhibitors have the potential to restore *PTEN* expression, which may present therapeutic benefit in breast cancer patients with *NOTCH* alterations. The development of compounds to target EZH2 in cancer remains to be an area of active interest, and EZH2-targeting compounds have been published displaying increased specificity and potency, including novel EZH2 degraders^58^. Further preclinical studies should include these improved inhibitors and degraders, as the effects on tumor cell viability in vitro and in vivo may be improved. Even though EZH2 can act as a tumor suppressor in certain tissues, EZH2 appears to act exclusively as an oncogene in breast cancer^59^. Our results and others^23^ show that γ-secretase inhibitors could also be a viable approach to restore PTEN expression in a subset of *NOTCH1/2*-altered patients that still harbor the γ-secretase cleavage site, either alone or in combination with EZH2 inhibitors.

Importantly, therapies that specifically aim to restore PTEN expression represent a largely unexplored strategy to boost tumor suppressor signaling. The strategies used in this study to restore *PTEN* expression could be relevant to other tumor suppressors in breast cancer, such as RB1.

## Methods

### Cell culture

Cell lines were purchased from ATCC. ATCC authenticates cell lines using several methods, including DNA fingerprinting. Cell lines were further authenticated in 2015 by LabCorp using a short tandem repeat method. Cell lines were tested quarterly for mycoplasma, and tested negative throughout the period of this study as determined by the Lonza Kit (LT07-418). MDA-MB-435S was used in Fig. 1 as part of a large panel of breast cancer cell lines used to measure PTEN expression levels. This line was selected to be part of this panel because it was wild-type for PTEN, and since the interpretation of this figure relies on many other cell lines, not only this one, we assume it is safe to include (especially since our cell lines have been validated).

All cells were cultured at 37 °C and 5% CO_2_. MCF10A cells were cultured in 50/50 DMEM/Ham’s F-12 media with 5% horse serum (Gibco 16050-122), 1X penicillin/streptomycin (Corning 30-002-Cl), 20 ng/ml of EGF (Peprotech AF-100-15), 10 µg/ml insulin (Sigma I9278), 0.5 mg/ml hydrocortisone (Sigma H0888), and 100 ng/ml cholera toxin (Sigma c8052). hMEC-hTERT cells were cultured in MEGM Complete media (CC-3051A & CC-4009). SUM-159 cells were cultured in 1X Ham’s F-12 media with 5% fetal bovine serum, 1X penicillin/streptomycin, 10 µg/ml insulin (Sigma I9278), and 0.5 mg/ml hydrocortisone (Sigma H0888). MDA-MB-157, BT-20, HS758T, MDA-MB-231, and MDA-MB-175-VII were cultured in 1X DMEM with 10% fetal bovine serum (Atlanta Biologicals S11150) 1X penicillin/streptomycin. HCC-1187, HCC-1143, HCC-1954, and HCC-2218 were cultured in 1X RPMI with 10% fetal bovine serum and 1X penicillin/streptomycin. Cells were split using 0.25% trypsin (Corning 25-053-Cl) before they reached full confluence and media was changed every 3–4 days. Corning Cellgro Media product information is as follows, DMEM: 10-013-CV, RPMI: 10-040-CV, 50/50 DMEM/Ham’s F-12: 10-090-CV, Ham’s F-12: 10-080-CV.

#### UNC1999

(Cayman 1431612-23-5) was used at indicated concentration for the indicated time periods. Control is treatment with equal volume of DMSO.

#### Compound E (CompE)

(Cayman 15579) was used at indicated concentration for the indicated time periods. Control is treatment with equal volume of DMSO.

### Human tissue samples

De-identified breast tissue samples used for Nanostring were distributed by the Tumor Bank in the Herbert Irving Comprehensive Cancer Center Molecular Pathology Shared Resource. De-identified breast tissue samples used for IHC, as well as H&E stains were distributed by the Biorepository and Pathology core at Icahn School of Medicine at Mount Sinai. All samples were considered non-human subject research by the IRBs of each institution.

### Immunohistochemistry

IHC was performed on formalin-fixed paraffin-embedded blanks from breast cancer cases from MSSM. Staining for EZH2 was performed and validated at the Molecular Cytology Core at MSKCC. Staining for PTEN was performed at Mount Sinai using the Leica-BOND automated IHC stainer. H&E slides for each case were provided with the blanks by MSSM. QurPath software was used for the analysis of IHC stain intensity quantification for PTEN and EZH2^60^.

#### Antibodies

EZH2 (Roche-SP219), PTEN (CST-138G6).

### Purification of epithelial cells from breast tissue

The protocol followed for purification of organoids from breast tissue has been previously published^33^, where we followed this protocol exactly. Samples were normal mammoplasty specimen from healthy patients. Further purification of epithelial cells from organoid preparations was performed using CELLection™ Epithelial Enrich Dynabeads^®^ (Thermo 16203, manufacturer’s protocol).

### Stable knockdowns

*Prepackaged viral particles containing shRNA (Sigma-Aldrich MISSION*^*®*^ *lentiviral transduction particles, SHCLNV):*

EZH2 shRNA#1: TRCN0000286227

EZH2 shRNA#2: TRCN0000286290

NOTCH2 shRNA#1: TRCN0000262587

NOTCH2 shRNA#2: TRCN0000282338

Negative Control (pKLO.1-puro non-target): SHC016V

*All shRNAs were expressed in the pKLO.1 vector backbone.

T25 flasks of cells (~30% confluent) were infected with indicated viral particles in in the presence of 12 μg/mL polybrene, and 2 μg/mL of puromycin (Sigma P8833) was used to select for infected cells. Used an MOI of one viral particle per cell.

### Luciferase reporter assay

Cells were seeded at 2 × 10^5^ cells/well of Falcon six-well dishes. The transfections were carried out the following day using Lipofectamine (18324-020) and Plus (11514-015) reagents according to the manufacturer’s instructions. The cells were harvested 24 h later using reagents supplied by the Dual-Luciferase^®^ Reporter Assay System (Promega E1910). Luciferase expression is normalized to Renilla activity, and was calculated as a fold change from the Pgl3-1 plasmid. The luciferase assays were performed as specified by the manufacturer’s instructions and were quantitated using a TD-20e Luminometer (Turner).

#### Luciferase plasmid

The pGL3 basic reporter vector was used (as described above). See below for the sections of the *PTEN* promoter that were cloned into the pGL3 vector, including the restriction sites that flank each section. These constructs were made by S. Nagase in the Parsons Laboratory^23^ and were sequenced before performing this experiment.

### qRT-PCR

RNA was prepared using the QiaShredder (79654) followed by the Qiagen RNeasy Kit (74104). cDNA was synthesized using the SuperScript Reverse Transcriptase II kit (Thermo 18064-014). The Applied Biosystems 7500 Fast Quantitative Realtime PCR System was used according to manufacturers’ protocol using Fast SYBR Green Master Mix (Thermo 4385612). All qRT-PCR values were normalized to GAPDH. Primer sequences are presented in Table 1.

**Table 1:** qRT-PCR primers.

Gene target	Sequence
PTEN-For	CCAGTCGCTGCAACCATC
PTEN-Rev	CTTCTTCTGCAGGATGGAAATG
ATAD1-For	AGTTGCCCAGGAAACTGATG
ATAD1-Rev	GTTGAACAGGCCGAATTTCA
EZH2-For	TTGTTGGCGGAAGCGTGTAAAATC
EZH2-Rev	TCCCTAGTCCCGCGCAATGAGC
NOTCH2-For	AACCTTCATGAAATGCAGCC
NOTCH2-Rev	CTGGAGACACAATGTGGTGG
NOTCH2-SEC22B-For	GGGTATAACTGTTGTCGCGG
NOTCH2-SEC22B-Rev	GAGTGAAACCTTCAGGCAGC
HES-1-For	CTGGAAATGACAGTGAAGCACCT
HES-1-Rev	ATTGATCTGGGTCATGCAGTTG
HEY-1-For	TGGATCACCTGAAAATGCTG
HEY-1-Rev	CGAAATCCCAAACTCCGATA
GAPDH-For	TCACCAGGGCTGCTTTTAAC
GAPDH-Rev	AATGAAGGGGTCATTGATGG

The temperature program was as follows:

Initial denaturation: 95 °C 20 s

40 cycles: 95 °C 3 s, 60 °C 30 s

### NanoString nCounter

Breast biopsies maximized for epithelial content were used to prepare RNA for Nanostring experiments. NanoString experiments were performed by the NanoString core at Icahn School of Medicine at Mount Sinai using probes for each gene pre-designed and validated by Nanostring. Total counts for each probe were obtained, and were normalized to the housekeeping genes (geometric mean of probe counts for all housekeeping genes) for each sample. See Supplementary Data 1 for NanoString probeset. The PTEN probes were located in the 3′UTR of *PTEN* because it contains unique regions from the PTEN pseudogene, *PTENP1*.

### Immunoblotting

Cells were lysed in 2× sample buffer (125 mM Tris-HCl at pH 6.8, 10% βME, 2% SDS, 20% glycerol, 0.05% Bromophenol Blue, 8 M urea). Protein lysates were loaded into 4–20% TRIS-glycine gels and resolved by electrophoresis. Samples were then blotted on PVDF membrane (Millipore IPVH00010) using the wet transfer technique (Invitrogen). Membranes were blocked in 5% milk-TBST for 1 h, washed in TBST for 10 min, and incubated in primary antibody in 5% milk-TBST or 5% BSA-TBST at 4 °C for 16 h. Membranes were rinsed (3 × 6 min) in TBST and incubated in horseradish peroxidase-conjugated secondary antibodies in 5% milk-TBST for 1 h and rinsed again in TBST (3 × 6 min). Membranes were visualized using the chemiluminescence system (Thermo 34080, 37075) on autoradiography film (Denville E3018).

#### Primary antibodies

Vinculin (Sigma V9131, 1:10,000), β-actin (Sigma A5316, 1:10,000), PTEN (138G6, CST 9559 1:1000), EZH2 (Active Motif 39901, 1:1000), and HES-1 (H-140, SC-25392. 1:300).

#### Secondary antibodies

Mouse (Thermo 31432, 1:5000), Rabbit (Thermo 31460, 1:5000).

### Chromatin immunoprecipitation (ChIP-qPCR)

ChIP assays were performed as previously described^61^. In summary, cells were cross-linked in 1% formaldehyde (J.T. Baker 2106-01) for 5 min on ice. After quenching with glycine, the cells were harvested in 1× PBS containing 1× protease inhibitor cocktail (Sigma P8340) and pelleted. For ChIP-qPCR, cells were sonicated for 20 min (30 s on, 30 s off) on the Diagenode Bioruptor Twin (UCD-400) sonicator at 4 °C. Lysates were precleared for 1 h with Protein A Agarose/Salmon Sperm DNA beads (Emdmillipore 16-157). Precleared lysates were then incubated with 7 μg of antibody overnight at 4 °C. Samples were then incubated with beads (same as preclear) for at least 2 h at 4 °C and beads were repeatedly washed. The Protein-DNA complexes were eluted, crosslinks were reversed, and DNA was purified using phenol/chloroform extraction followed by sodium acetate/ethanol precipitation. % input was calculated and normalized as a fold change from IgG. Antibodies: IgG (sc-2025), EZH2 (Active Motif 39901), HES-1 (H-140, SC-25392), H3K27Me3 (Millipore 07-449). ChIP-qPCR primers are presented in Table 2.

**Table 2:** ChIP-qPCR primers.

Name	Sequence	Hg19 Loc. (Chr10)
Site 1-For	GAGAGATTTGGGACATGGGA	89618828
Site 1-Rev	GCGCTACTGTGGGTCATACA	89618913
Site 2-For	GCACCCTTGTTTCATTTGCT	89620304
Site 2-Rev	CCCTGGAGCCTACCCTAAGT	89620393
Site 3-For	GCTCAGGGGTAGTGACTGGA	89621491
Site 3-Rev	TTGAGGGTATCTCCTGCTGC	89621567
Site 4-For	CGGGCTTCAAAAGTTAGTGG	89625500
Site 4-Rev	CCCCATCCCTAATCAAAACC	89625583
Site B-For	GTGATGTGGCGGGACTCTTTAT	89623313
Site B-Rev	CTCTCATCTCCCTCGCCTGAG	89623472

### Proliferation assay

Cells (Empty Vector and *EZH2* shRNA#1 and *EZH2* shRNA#2) were plated at 8000 cells/well (HCC-1187) or 2000 cells/well (MDA-MB-231) in 96-well tissue culture plates (Corning 3595) full media. Cells were allowed to grow for the indicated number of days. The Essen BioScience IncuCyte^®^ ZOOM Live-Cell Analysis System took phase-contrast images in triplicate wells every 6 h. The IncuCyte^®^ software package was used to estimate confluence at each time point.

### Data from the cancer genome atlas (TCGA)

TCGA data shown is from the invasive breast cancer dataset (818 cases)^34^ including pre-analyzed mutation, RNA-seq, and reverse phase protein array data was downloaded from the cBioPortal^30,31,34^, except for the data contained in Supplementary Fig. 1a, e, which are from the breast invasive carcinoma TCGA Firehose Legacy cohort (1108 cases). All complete tumors were used for any given analysis, and the number of cases used is indicated for each analysis.

### Co-expression analysis

Genes that were co-expressed with PTEN in an invasive breast cancer dataset published by TCGA were analyzed using the co-expression tool in cBioPortal^30,31,34^. Pearson (*r*) scores were provided by cBioPortal and *P* values were calculated using a two-tailed *t*-test (*n* = 818). *P* values were adjusted for multiple comparisons using the Benjamini and Yekutieli method^62^. We established a significance cutoff defined by a *P* value (*P* < 1.0 × 10^−3^).

### Gene set enrichment analysis (GSEA)

Enrichment of the list of invasive breast cancer cases^34^ (pre-ranked from high to low EZH2 expression by RNA-seq z-score) for cases with mutations in the *TP53* gene was quantified using the GSEA package^63^.

### Analysis of chromatin landscape of *PTEN* promoter

Supplementary Fig. 2 was generated using R. Details and code can be accessed at the following link, https://github.com/TiphaineCMartin/Regulation_PTEN_KyriePappas2018. Various previously published and publicly available datasets were used for this analysis from COSMIC (Release v84, February 13, 2018, cancer.sanger.ac.uk), ENCODE (ChromHMM hMEC GEO sample accession: GSM936084, DNAse-seq hMEC sample accession: GSM736634, MCF7 sample accession: GSM736588), ENSEMBL (Release 91 of GRCh37.13), and others^15,35,64–67^.

### Statistics and reproducibility

No statistical methods were used to determine sample size, and experiments were not randomized. The experimenters were not blinded. Replicates and sample sizes were determined for each experiment based on feasibility within method being used. Aside from traditional Mann–Whitney, Pearson correlation test, and student *t* tests to compare data sets, statistical methods were used in order to make appropriate multiple comparisons of data (following one-way or two-way ANOVA as indicated in figure legends). Graphpad Prism 6 was used to make these simple predetermined statistical comparisons. When multiple cell lines were used for an experiment, corrections for multiple comparisons were performed on the combined data.

#### Dunnett’s multiple comparisons correction

Used for comparing all samples to a control sample, but not for comparing the non-control samples to one another.

#### Sidak’s multiple comparisons correction

Used when specific multiple comparisons are pre-selected.

#### Tukey’s multiple comparisons correction

Used when all pairwise comparisons are performed.

#### Benjamini and Yekutieli correction (^62^)

Used for Pearson correlation test.

We also use the Logrank nonparametric test to determine the statistical difference between Kaplan–Meier survival distributions.

### Reporting summary

Further information on research design is available in the Nature Research Reporting Summary linked to this article.

## Supplementary information

Supplementary Information

Description of Additional Supplementary Files

Supplementary Data 1

Supplementary Data 2

Supplementary Data 3

Supplementary Data 4

Supplementary Data 5

Reporting Summary

## Data Availability

Data analyzed in Supplementary Fig. 2 can be found at https://github.com/TiphaineCMartin/Regulation_PTEN_KyriePappas2018. Source data for all figures can be found in Supplementary Data 5. All other data generated or analyzed during this study are included in this published article (and its Supplementary Information files).

## Code availability

All custom scripts have been made available at https://github.com/TiphaineCMartin/Regulation_PTEN_KyriePappas2018.
