# Supplementary material for: NOTCH and EZH2 collaborate to repress PTEN expression in breast cancer
Source: Commun Biol. 2021 Mar 9;4:312. doi: 10.1038/s42003-021-01825-8 (PMC7943788; doi:10.1038/s42003-021-01825-8)
Supplement: Supplementary file 1 — Supplementary Information [file 42003_2021_1825_MOESM1_ESM.pdf]

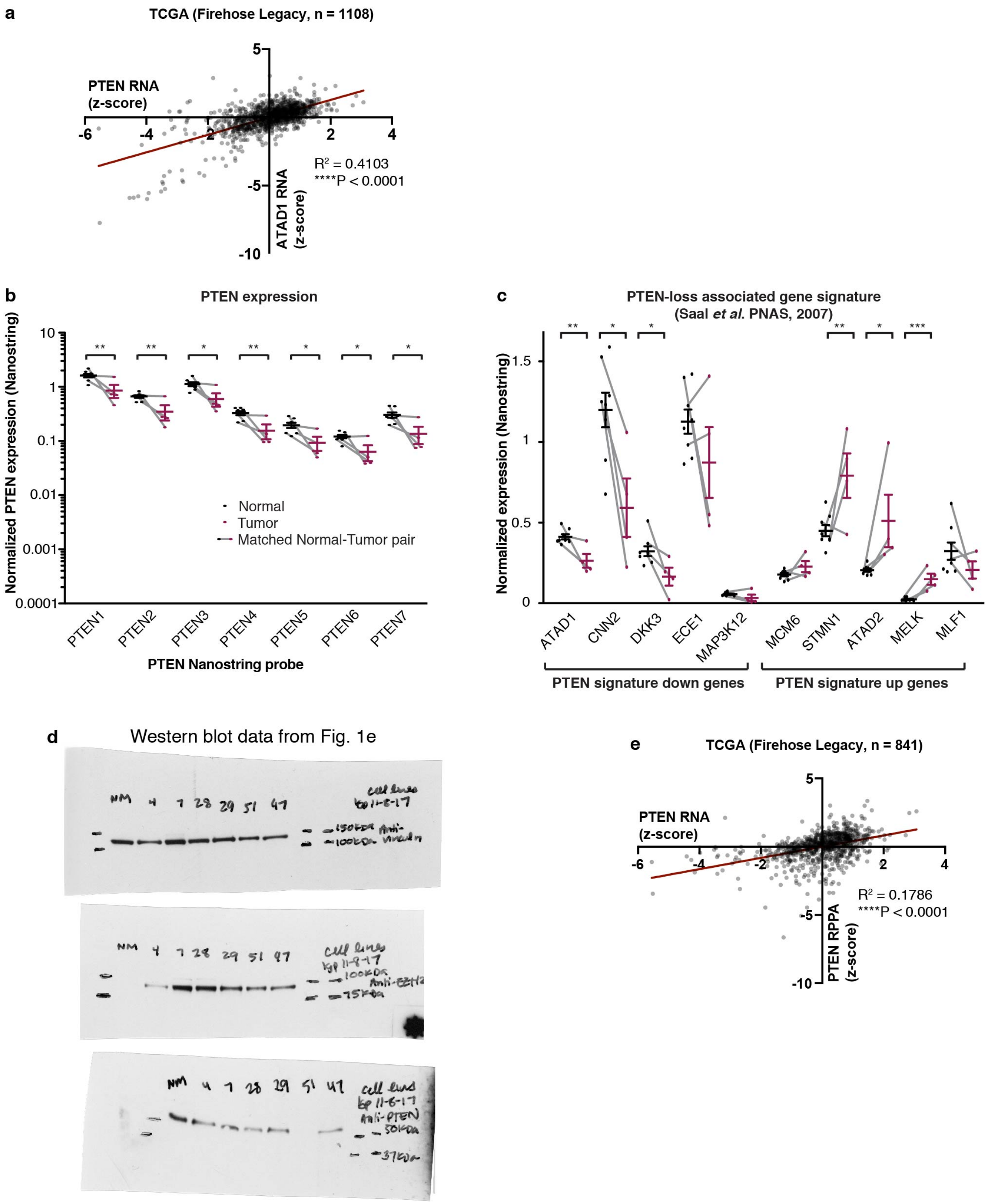
a
TCGA (Firehose Legacy, n = 1108)
PTEN RNA
(z-score)
ATAD1 RNA
(z-score)
R^2 = 0.4103
****P < 0.0001
b
PTEN expression
Normalized PTEN expression (Nanostring)
Normal
Tumor
Matched Normal-Tumor pair
PTEN1
PTEN2
PTEN3
PTEN4
PTEN5
PTEN6
PTEN7
PTEN Nanostring probe
c
PTEN-loss associated gene signature
(Saal et al. PNAS, 2007)
Normalized expression (Nanostring)
ATAD1
CNN2
DKK3
ECE1
MAP3K12
MCM6
STMN1
ATAD2
MELK
MLF1
PTEN signature down genes
PTEN signature up genes
d
Western blot data from Fig. 1e
e
TCGA (Firehose Legacy, n = 841)
PTEN RNA
(z-score)
PTEN RPPA
(z-score)
R^2 = 0.1786
****P < 0.0001

Supplementary Figure 1: Transcriptional repression of *PTEN* and associated poor-prognosis gene expression signature is present in tumor but not adjacent normal breast samples. **a** Scatter plot showing PTEN and ATAD1 levels by RNA-seq (z-score) in breast cancer cases (n= 841). Linear regression was performed; trendline, R-squared (R^2), and P-value displayed on chart [1,2]. Nanostring was used to measure transcript levels of **b** PTEN and **c** various members of the PTEN-loss associated gene signature [3] in 4 normal-tumor pairs and 4 additional normal breast tissue biopsy samples. Normal shown in black, tumor in red, N-T pairs are connected by gray line. See Supplementary Spreadsheet S1 for Nanostring probes. Error Bars are mean ± s.e.m. Significance: 2-tailed t-test, Holm-Sidak correction for multiple comparisons. (***P<0.001, **P<0.01, *P<0.05) **d** Uncropped immunoblot images from Figure 1e. Vinculin is loading control. **e** Scatter plot showing PTEN transcript levels by RNA-seq and PTEN protein levels by RPPA in breast cancer cases (n= 841). Linear regression was performed; trendline, R-squared (R^2), and P-value displayed on chart [1,2].

a

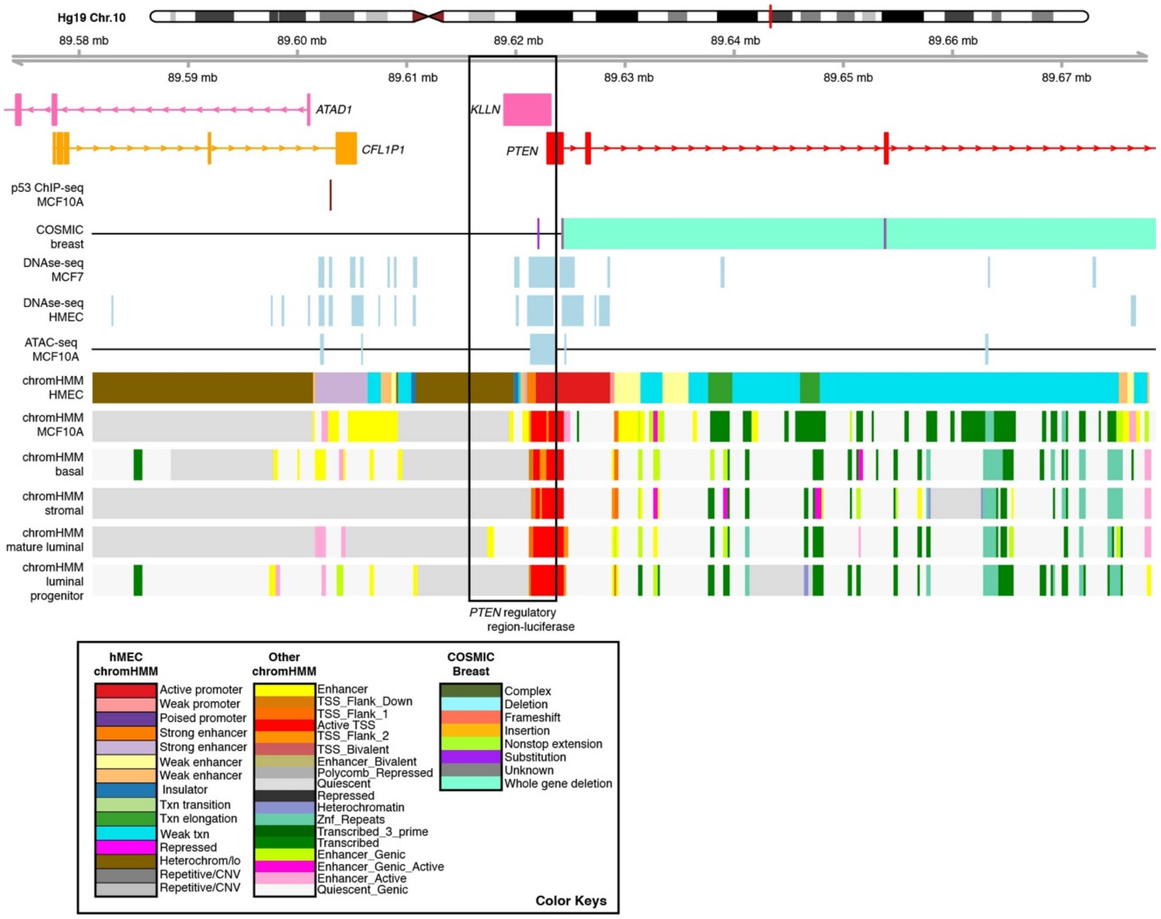

b

Western blot data from Fig. 2d

Western blot data from Fig. 2e-f

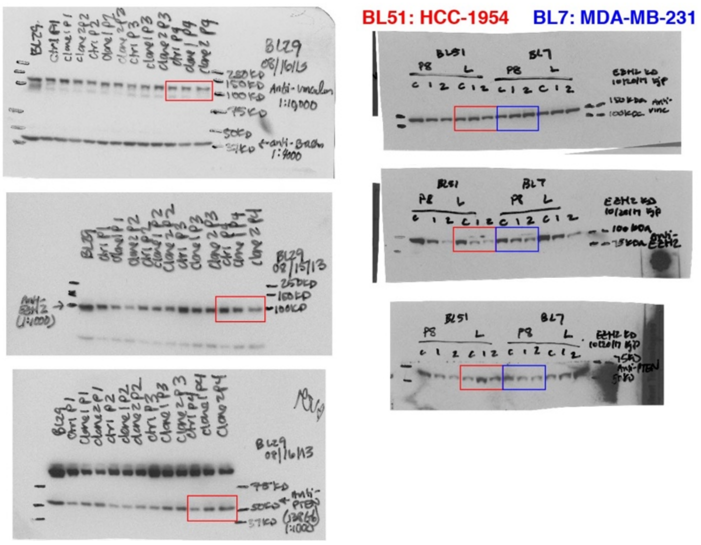

Supplementary Figure 2: Transcriptional landscape of the *PTEN* promoter and regulatory region. Characterization of the chromatin accessibility and transcriptional landscape of the *PTEN* promoter and upstream regulatory region including p53 ChIP-seq in MCF10A non-tumorigenic mammary epithelial cells, COSMIC genetic alterations in breast cancer, DNAse-seq in MCF7 breast cancer cells and non-tumorigenic human mammary epithelial cells (HMEC), and chromHMM in HMEC and MCF10A cells (non-tumorigenic cell lines), and in basal, stromal, and luminal (mature and progenitor) breast cells. *PTEN* regulatory region analyzed in the luciferase assay in Fig. 2 is highlighted (black box). See Materials and Methods for additional detail and dataset references. **b** Uncropped blot images from Figure 2d-f. Vinculin is loading control.

a

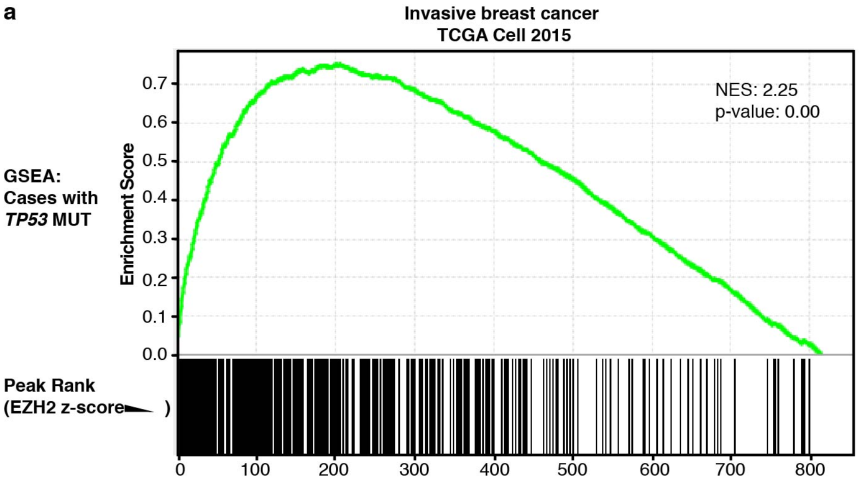

b

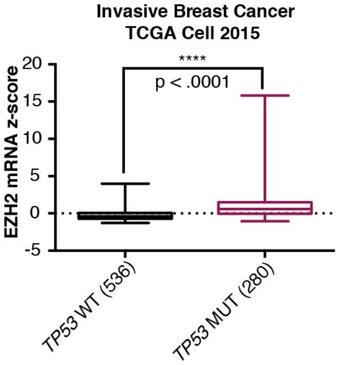

c

Cell Line	*TP53* Status
HCC-1187	MUT
HCC-1954	MUT
HCC-2218	MUT
MDA-MB-231	MUT
HCC-1143	MUT
MDA-MB-175-VII	WT

d Western blot data from Fig. 5b

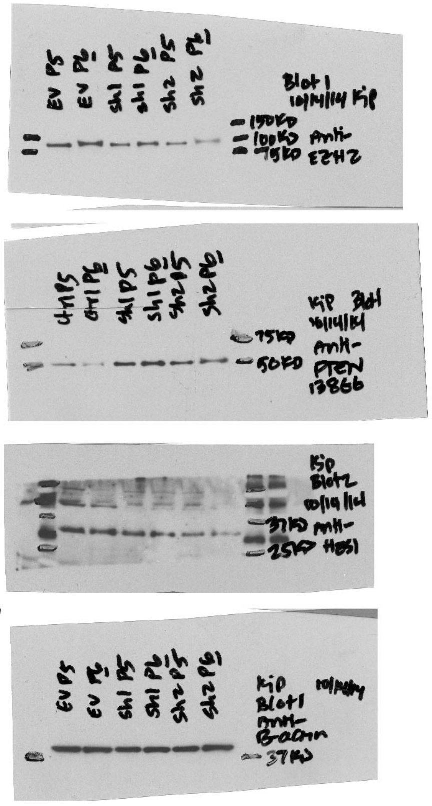

Western blot data from Fig. 5e

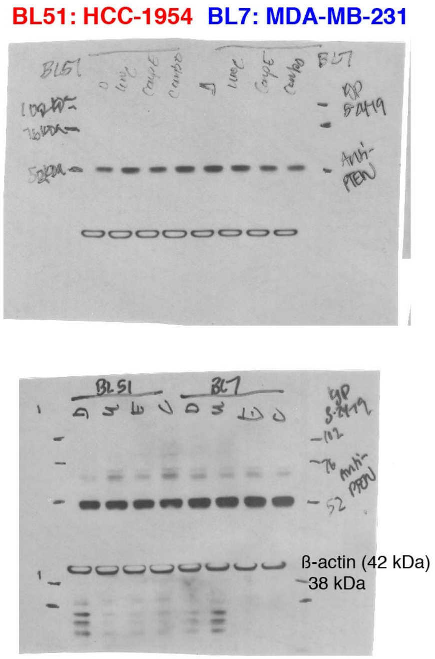

34

Supplementary Figure 3: *TP53* mutation associates with increased expression of EZH2 in breast cancer, but does not associate with restoration of PTEN expression in response to EZH2 inhibition. a Gene set enrichment analysis (GSEA) was performed on a list of 816 invasive breast cancer cases [4] pre-ordered for EZH2 RNA-seq z-score for mutation of *TP53*. Normalized enrichment score (NES) and p-value are displayed. **b** Boxplots of EZH2 expression in *TP53*-mutant versus wild-type case. Significance: Mann-Whitney test. Number of cases indicated for each group (total n = 816). (****P<0.0001) **c** *TP53* mutation status of cell lines used in knockdown and EZH2 inhibitor studies (cancer.sanger.ac.uk) [5]. **d** Uncropped blot images from Figure 5b and e. β-actin is loading control.

Cell Line	Subtype	ER	PR	HER2
HS758T	TNBC	Neg	Neg	Neg
HCC-202	HER2	Neg	Neg	Pos
HCC-1187	TNBC	Neg	Neg	Neg
HCC-1143	TNBC	Neg	Neg	Neg
HCC-1599	TNBC	Neg	Neg	Neg
SUM-159	TNBC	Neg	Neg	Neg
HCC-2218	HER2	Neg	Neg	Pos
MDA-MB-330	LUMB	Pos	Neg	Pos
HCC-1954	TNBC	Neg	Neg	Neg
BT-474	LUMB	Pos	Pos	Pos
HCC-1806	TNBC	Neg	Neg	Neg
ZRC75-30	LUMB	Pos	Neg	Pos
L56BR	TNBC	Neg	Neg	Neg
MDA-MB-361	LUMB	Pos	Pos/Neg*	Pos
HCC-1419	LUMB	Pos/Neg*	Neg	Pos
MDA-MB-157	TNBC	Neg	Neg	Neg
DU-4475	TNBC	Neg	Neg	Neg
UACC-812	LUMB	Pos	Pos/Neg*	Pos
HCC-38	TNBC	Neg	Neg	Neg
UACC-893	HER2	Neg	Neg	Pos
BT-483	LUMA	Pos	Pos/Neg*	Neg
MDA-MB-231	TNBC	Neg	Neg	Neg
SUM-1315	TNBC	Neg	Neg	Neg
HCC-1428	LUMA	Pos	Pos	Neg
MDA-MB-175-VII	LUMA	Pos	Neg	Neg
BT-20	TNBC	Neg	Neg	Neg
HCC-1569	HER2	Neg	Neg	Pos
MDA-MB-435-S	TNBC	Neg	Neg	Neg

Supplementary Table 1: Molecular characteristics of breast cancer cell lines [6,7]. Table contains (left to right) cell line name, breast cancer subtype, immunohistochemical status of estrogen receptor (ER), progesterone receptor (PR), and Her2 listed as positive (Pos) or negative (Neg). *Cell lines listed as Pos/Neg have published values that are contradictory.

BlockID	PatientID	N/T	ER	PR	Her2
2656	1589	N	n/a	n/a	n/a
2568	1590	N	n/a	n/a	n/a
8207	3937	N	n/a	n/a	n/a
8216	3931	N	n/a	n/a	n/a
2611	1601	N	n/a	n/a	n/a
2614	1602	N	n/a	n/a	n/a
3206	1978	N	n/a	n/a	n/a
3194	1972	N	n/a	n/a	n/a
2613	1601	T	Pos/100	Pos/50	Neg/0
2615	1602	T	Neg/00	Neg/00	Pos/3+
3207	1978	T	Pos/80	Pos/30	Neg/1+
3196	1972	T	Neg/00	Neg/00	Pos/3+

Supplementary Table 2: Molecular characteristics of breast cancer tumor samples from CUMC tumor bank. Table contains (left to right) BlockID and PatientID, normal (adjacent) or tumor sample (N/T), and clinical immunohistochemistry scoring and status of ER, PR, and Her2 (only available for tumor samples). Score is numerical, status is Pos or Neg.

Supplementary References:

1 Cerami, E. *et al.* The cBio cancer genomics portal: an open platform for exploring multidimensional cancer genomics data. *Cancer discovery* **2**, 401-404, doi:10.1158/2159-8290.CD-12-0095 (2012).

2 Gao, J. *et al.* Integrative analysis of complex cancer genomics and clinical profiles using the cBioPortal. *Sci Signal* **6**, pl1, doi:10.1126/scisignal.2004088 (2013).

3 Saal, L. H. *et al.* Poor prognosis in carcinoma is associated with a gene expression signature of aberrant PTEN tumor suppressor pathway activity. *Proceedings of the National Academy of Sciences* **104**, 7564-7569, doi:10.1073/pnas.0702507104 (2007).

4 Ciriello, G. *et al.* Comprehensive Molecular Portraits of Invasive Lobular Breast Cancer. *Cell* **163**, 506-519, doi:10.1016/j.cell.2015.09.033 (2015).

5 Forbes, S. A. *et al.* COSMIC: somatic cancer genetics at high-resolution. *Nucleic Acids Res* **45**, D777-D783, doi:10.1093/nar/gkw1121 (2017).

6 Dai, X., Cheng, H., Bai, Z. & Li, J. Breast Cancer Cell Line Classification and Its Relevance with Breast Tumor Subtyping. *J Cancer* **8**, 3131-3141, doi:10.7150/jca.18457 (2017).

7 Johannsson, O. T. *et al.* Characterization of a novel breast carcinoma xenograft and cell line derived from a BRCA1 germ-line mutation carrier. *Lab Invest* **83**, 387-396 (2003).
