# Supplementary material for: NOTCH and EZH2 collaborate to repress PTEN expression in breast cancer
Source: Commun Biol. 2021 Mar 9;4:312. doi: 10.1038/s42003-021-01825-8 (PMC7943788; doi:10.1038/s42003-021-01825-8)
Supplement: Supplementary file 2 — Description of Additional Supplementary Files [file 42003_2021_1825_MOESM2_ESM.pdf]

Description of Additional Supplementary Files

File name: Supplementary Data 1
Description: NanoString nCounter probeset of probes including seven PTEN mRNA-specific probes, PTEN-loss associated signature genes (up and down), and housekeeping genes. Table contains transcript name, hg19 genomic location of nCounter probe (strand, chromosome, start, end), target sequence, melting temperature of capture probe (Tm_CP) and reporter probe (Tm_RP), and probe category. Signature genes indicate 'up' or 'down' for the change in transcript level upon PTEN-loss in breast tumors. For housekeeping genes, the geometric mean of the expression is calculated for normalization.

File name: Supplementary Data 2
Description: Correlation analysis of genes co-expressed with PTEN by RNA-seq. Analysis of genes co-expressed with PTEN including gene name, cytoband, and Pearson correlation scores (r-values) in normalized RNA-seq data. P-values were calculated using a 2-tailed t-test. Cohort includes 818 breast cancer cases. P-values adjusted for multiple comparisons by the Benjamini and Yekutieli method.

File name: Supplementary Data 3
Description: Molecular characteristics of breast cancer tumor samples from Icahn School of Medicine at Mount Sinai (MSSM). Table contains (left to right) Biorepository ID, clinical immunohistochemistry scoring of ER, PR, and Her2 by IHC (Score is numerical, and % positivity, if available), HER2 FISH if available, and TNBC (Y/N). NC means data is not clear. Table also contains manual scoring (0-3+) for PTEN and EZH2 immunohistochemistry including score for tumor (T), andjacent normal breast ducts (N), average score for both T and N, the delta score between avg T and avg N (delta T-N). Multiple scores for a single case indicate sections of different staining intensity within slide scan.

File name: Supplementary Data 4
Description: Correlation analysis of genes co-expressed with RB1 by RNA-seq. Analysis of genes co-expressed with RB1 including gene name, cytoband, and Pearson correlation scores (r-values) in normalized RNA-seq data. P-values were calculated using a 2-tailed t-test. Cohort includes 818 breast cancer cases. P-values adjusted for multiple comparisons by the Benjamini and Yekutieli method.

File name: Supplementary Data 5
Description: Source data for figures
