# Supplementary material for: NOTCH and EZH2 collaborate to repress PTEN expression in breast cancer
Source: Commun Biol. 2021 Mar 9;4:312. doi: 10.1038/s42003-021-01825-8 (PMC7943788; doi:10.1038/s42003-021-01825-8)
Supplement: Supplementary file 8 — Reporting Summary [file 42003_2021_1825_MOESM8_ESM.pdf]

nature research

Corresponding author(s): Ramon Parsons

Last updated by author(s): 1-26-2021

Reporting Summary

Nature Research wishes to improve the reproducibility of the work that we publish. This form provides structure for consistency and transparency in reporting. For further information on Nature Research policies, see Authors & Referees and the Editorial Policy Checklist.

Statistics

For all statistical analyses, confirm that the following items are present in the figure legend, table legend, main text, or Methods section.

n/a	Confirmed	
☐	☒	The exact sample size (n) for each experimental group/condition, given as a discrete number and unit of measurement
☐	☒	A statement on whether measurements were taken from distinct samples or whether the same sample was measured repeatedly
☐	☒	The statistical test(s) used AND whether they are one- or two-sided<br>*Only common tests should be described solely by name; describe more complex techniques in the Methods section.*
☐	☒	A description of all covariates tested
☐	☒	A description of any assumptions or corrections, such as tests of normality and adjustment for multiple comparisons
☐	☒	A full description of the statistical parameters including central tendency (e.g. means) or other basic estimates (e.g. regression coefficient) AND variation (e.g. standard deviation) or associated estimates of uncertainty (e.g. confidence intervals)
☐	☒	For null hypothesis testing, the test statistic (e.g. F, t, r) with confidence intervals, effect sizes, degrees of freedom and P value noted<br>*Give P values as exact values whenever suitable.*
☒	☐	For Bayesian analysis, information on the choice of priors and Markov chain Monte Carlo settings
☒	☐	For hierarchical and complex designs, identification of the appropriate level for tests and full reporting of outcomes
☐	☒	Estimates of effect sizes (e.g. Cohen's d, Pearson's r), indicating how they were calculated

Our web collection on statistics for biologists contains articles on many of the points above.

Software and code

Policy information about availability of computer code

Data collection	Code availability: All custom scripts have been made available at https://github.com/TiphaineCMartin/Regulation_PTEN_KyriePappas2018.
Data analysis	Code availability: All custom scripts have been made available at https://github.com/TiphaineCMartin/Regulation_PTEN_KyriePappas2018.

For manuscripts utilizing custom algorithms or software that are central to the research but not yet described in published literature, software must be made available to editors/reviewers. We strongly encourage code deposition in a community repository (e.g. GitHub). See the Nature Research guidelines for submitting code & software for further information.

Data

Policy information about availability of data

All manuscripts must include a data availability statement. This statement should provide the following information, where applicable:

- Accession codes, unique identifiers, or web links for publicly available datasets
- A list of figures that have associated raw data
- A description of any restrictions on data availability

Data analyzed in Supplementary Figure 2 can be found at https://github.com/TiphaineCMartin/Regulation_PTEN_KyriePappas2018. Source data for all figures can be found in Supplementary Data 5. All other data generated or analyzed during this study are included in this published article (and its supplementary information files).

Code availability: All custom scripts have been made available at https://github.com/TiphaineCMartin/Regulation_PTEN_KyriePappas2018.

Field-specific reporting

Please select the one below that is the best fit for your research. If you are not sure, read the appropriate sections before making your selection.

☒ Life sciences ☐ Behavioural & social sciences ☐ Ecological, evolutionary & environmental sciences

For a reference copy of the document with all sections, see nature.com/documents/nr-reporting-summary-flat.pdf

Life sciences study design

All studies must disclose on these points even when the disclosure is negative.

Sample size	Sample sizes were chosen based on reagent, sample, and data availability. We generally attempted to perform in vitro studies in multiple cell lines, and human breast cancer patient datasets used in this study have exceedingly large sample sizes. No statistical test was used to determine sample size.
Data exclusions	n/a
Replication	Number of replicates performed are indicated in the figure legend for all relevant experiments. All replication studies were successful.
Randomization	n/a
Blinding	n/a. No experiments where blinding would be applicable were performed in this study.

Reporting for specific materials, systems and methods

We require information from authors about some types of materials, experimental systems and methods used in many studies. Here, indicate whether each material, system or method listed is relevant to your study. If you are not sure if a list item applies to your research, read the appropriate section before selecting a response.

Materials & experimental systems

n/a	Involved in the study	
☐	☒	Antibodies
☐	☒	Eukaryotic cell lines
☒	☐	Palaeontology
☒	☐	Animals and other organisms
☒	☐	Human research participants
☒	☐	Clinical data

Methods

n/a	Involved in the study	
☒	☐	ChIP-seq
☒	☐	Flow cytometry
☒	☐	MRI-based neuroimaging

Antibodies

Antibodies used	Primary Antibodies: Vinculin (Sigma V9131), β-actin (Sigma A5316), PTEN (138G6, CST 9559), EZH2 (Active Motif 39901), HES-1 (H-140, SC-25392).<br>Secondary Antibodies: Mouse (Thermo 31432), Rabbit (Thermo 31460).<br>Antibody use was not confined to a single lot number.
Validation	All antibodies used are commercially available. All companies are widely used and trusted for the production of antibodies and use extensive validation processes. Please see antibody validation information below.<br>CST: https://www.cellsignal.com/contents/our-approach/cst-antibody-validation-principles/ourapproach-validation-principles<br>Thermo: https://www.thermofisher.com/us/en/home/life-science/antibodies/invitrogen-antibody-validation.html<br>Sigma: https://www.sigmaaldrich.com/life-science/cell-biology/antibodies/antibody-validation.html<br>Active Motif: https://www.activemotif.com/antibody-development<br>scbt: https://www.scbt.com/scbt/browse/antibodies/_/N-med3ky

Eukaryotic cell lines

Policy information about cell lines

Cell line source(s)	Cell lines were purchased from ATCC.
Authentication	The ATCC authenticates cell lines using several methods, including DNA fingerprinting. Cell lines were further authenticated in 2015 by LabCorp using a short tandem repeat method.

Mycoplasma contamination	Cell lines were tested quarterly for mycoplasma, all tested negative throughout the period of this study as determined by the Lonza Kit (LT07-418).
Commonly misidentified lines<br>(See ICLAC register)	MDA-MB-435S was used in Figure 1 as part of a large panel of breast cancer cell lines used to measure PTEN expression levels. This line was selected to be part of this panel because it was wild-type for PTEN, and since the interpretation of this figure relies on many other cell lines, not only this one, we assume it is safe to include (especially since our cell lines have been validated).
